# Primary Carcinoma of the Fallopian Tube: A Review of a Single Institution Experience of 8 Cases

**DOI:** 10.1155/2014/630731

**Published:** 2014-02-13

**Authors:** Shakuntala P. Nanaiah, Praveen S. Rathod, Namrata N. Rajkumar, Rajshekar Kundargi, Anbukkani Subbian, Pallavi V. Ramachandra, Shobha Krishnappa, Abhilasha Narayan, Uma K. Devi, Bafna D. Uttamchand

**Affiliations:** ^1^Department of Gynaecologic Oncology, Kidwai Memorial Institute of Oncology, Dr. MH MariGowda Road, Bengaluru, Karnataka 560030, India; ^2^Department of Pathology, Kidwai Memorial Institute of Oncology, Dr. MH MariGowda Road, Bengaluru, Karnataka 560030, India

## Abstract

*Aims and Objectives*. To evaluate the clinicopathologic features, response to cytoreductive surgery and adjuvant platinum-based chemotherapy with or without paclitaxel. *Materials and Methods*. A retrospective observational study of 8 women with a histopathologic diagnosis of primary fallopian tube carcinoma (PFTC) from January 2000 to February 2013. *Results*. 4/8 (50%) of the women were in the early stage and an intraoperative frozen section was 100% effective in identifying fallopian tube carcinoma and then a staging laparotomy was performed. All 4/8 cases in the early stage had received and responded to single agent carboplatin and all are alive without clinical, radiological, or biochemical evidence of recurrence at the end of 2 years and the longest survivor has completed 13 years. Primary optimal cytoreductive surgery was achievable in 3/4 (75%) in advanced disease. All showed response to adjuvant paclitaxel and carboplatin (T+C), but all had succumbed to the disease following recurrence with mean progression-free survival of 19 months (range 15–21 months) and mean overall survival of 27 months (range 22–36 months). *Conclusion*. The pivotal role played by a frozen section in diagnosing PFTC which is rare needs to be reemphasized, therefore justifying a primary staging laparotomy in an early stage. Prolonged survival observed in this group following an optimum tailored adjuvant single agent carboplatin is worth noting.

## 1. Introduction

Primary fallopian tube carcinoma (PFTC) is rare and it accounts for only 0.3% to 1% of gynaecologic malignancies [1]. Close to 3000 women have been diagnosed as PFTC and described in the English medical literature [2–8]. International Federation of Obstetrics and Gynaecology (FIGO) published a staging system for PFTC in 1991 [9]. Alvarado-Cabrero et al. proposed a modified staging system that subclassified stages IA and IB according to the depth of invasion within the wall of the tube as (a) no invasion, (b) invasion into the lamina propria or invasion into muscularis. This modified system also classified tumors which were located in the fimbrial end (noninvasive tumours) as stage IF, based on the capability of these lesions to metastasize into the peritoneal cavity even when noninvasive. This modified staging system reported enhanced prognostic significance of staging as demonstrated in the study published in 1999 [10]. However, these modifications were never implemented by FIGO. The current FIGO staging system for fallopian tube cancers is still the same as the one published in 1991.

Paley et al. in 2001 [11] reported that, when the fallopian tubes were more carefully examined, confirmed that in situ and small, early invasive tubal carcinomas occurred in women with a genetic predisposition for the development of ovarian carcinoma. This led to fallopian tube carcinoma being included as part of the cancer spectrum associated with inherited BRCA mutations. Subsequently, Kurman and Shih in 2010 and 2011 [12, 13] had proposed that ovarian serous tumors arise from the implantation of epithelium (benign or malignant) from the fallopian tube. Endometrioid and clear cell ovarian tumors have been associated with endometriosis, which is regarded as the precursor of these tumors. Preliminary data suggest that mucinous and transitional (Brenner) tumors arise from transitional-type epithelial nests at the tubal-mesothelial junction by a process of metaplasia. But what needs to be questioned is that though fallopian tube is the proposed site of origin for serous subtype of epithelial ovarian cancers, per se fallopian tube cancers are very rare. Our institutional data was 0.11% (08/7200) cases of ovarian tumours.

Data is very heterogenous with respect to the details of methods of diagnosis, staging, grading, and adjuvant treatment [14, 15]. So when clinically challenged with this subtype of tumour, the literature search provided us with the data pertaining to staging and adjuvant chemotherapy as will be reviewed and discussed.

## 2. Material and Methods

This includes the following:

- is there a role for frozen section in proceeding with staging laparotomy in presumed early stage disease intraoperatively?
- role of adjuvant single agent carboplatin in early stage disease which is comprised of Stage IA, B, and C to Stage IIA, including all grades (G1, 2, and 3),
- the response to Cytoreductive surgery plus adjuvant paclitaxel with platinum-based chemotherapy in advanced stage disease which is comprised of Stage IIB–IVB and included all grades (G1, 2, 3),
- assess the progression-free and overall survival.

We reviewed the records of 8 women from January 2000 to February 2013 at Kidwai Memorial Institute of Oncology. Institutional review board had approved the study.

Intraoperative frozen section was performed in women with tubal thickening or gross evidence of a tubal mass (conditions like hydrosalphix, haematosalphinx, or simple cysts associated with or without adnexal torsion or a strong suspicion of fallopian tube tumour) by palpation during the surgical procedure and, if the frozen section revealed fallopian tube cancer, we proceeded with staging laparotomy which consisted of obtaining samples of peritoneal fluid washings from 5 sites (pouch of douglas, bilateral paracolic gutters, and bilateral subdiaphragmatic areas) in the peritoneal cavity following a saline wash in the absence of obvious ascites. A total abdominal hysterectomy, bilateral salphingooophorectomy, pelvic and para-aortic nodal dissection, and infracolic omentectomy were then performed.

In advanced stage disease, the aim was to achieve residual disease <1 cm termed as primary optimal debulking surgery. Surgical procedures included total abdominal hysterectomy, bilateral salphingooophorectomy, tumor debulking, total omentectomy, bilateral pelvic lymphnode dissection, and para-aortic node dissection. All the pathological specimens were reviewed by the gynaecologic oncopathologists. Histopathological analysis was performed on all specimens as described in WHO Manual [16]. Final stage allotment was based on surgicopathologic criteria for fallopian tube carcinoma published by FIGO 1992 [9].

Paclitaxel dose of 175 mg/M^2^ was administered over 3 hours of infusion followed by Carboplatin which was calculated using Calverts formula and area under curve (AUC-6-7).

Response to chemotherapy and surgery was evaluated by computed tomography or ultrasound according to World Health Organisation (WHO) criteria in patients with measureable disease [17].

Patients with tumors assessed by CT scan (≥10 mm) or by ultrasonography (≥20 mm) were classified as measurable disease. Nonmeasurable disease included cystic lesions and ascites. Patients with measurable disease after primary cytoreductive surgery were assessed for objective response. Pelvic and abdominal CT scan or USG and chest X-ray were repeated after the third and the sixth treatment courses.

Progression-free survival was calculated from the date of registration to the date of radiological or clinical appearance of the disease confirmed histologically or by cytology following treatment.

Overall survival was calculated from the date of registration to date of death or the last followup.

Statistical analysis was performed using SPSS base 10.0. Survival estimates were obtained via Kaplan-Meier method. The log rank test that is used to assess the prognostic importance of histopathological characteristics, therapeutic modalities, and *P* values < 0.05 considered statistically significant could not be obtained due small number of patients.

## 3. Results

The mean age was 55.5 years (range 40–68 years). Mean parity was 2 (range 0–4) and 5/8 (62.5%) of the women were postmenopausal with a mean postmenopausal period of 6 years (2–16 years).

Abdominal pain with distension was the commonest complaint encountered in 7/8 (87.5%), the classical triad of pelvic pain, mass abdomen, and episodes of watery vaginal discharge relieving the pain abdomen was noted in 1/8 (12.5%) of women, Table 1 (case no. 2). Irregular bleeding and vaginal discharge were noted in 2/8 (25%) of the women. 75% had a ECOG score of 0-1, Table 2.

**Figure 1:**
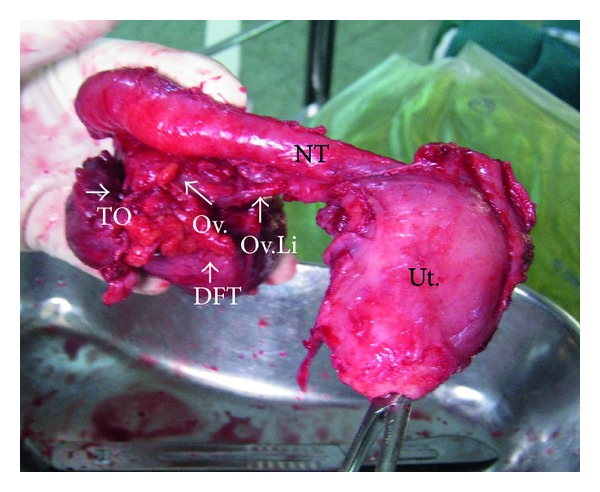
Showing a normal uterus (Ut.), normal medial portion of the fallopian tube (NT), thickened and dilated to form a retort shape along with growth (DFT-G), and occluding the tubal ostia due to growth (TO). Normal ovary (Ov.) and ovarian ligament (Ov.Li.) are also seen.

**Figure 2:**
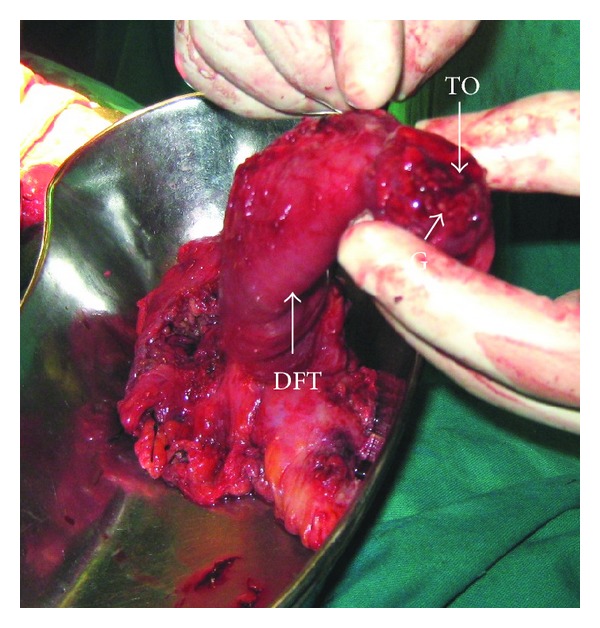
Tuboovarian mass showing dilated and distended fimbrial end and occlusion of the tubal ostia (TO) due to growth. Distended fallopian tube with growth (DFT), also seen is involved ovarian tissue below (Ov.).

**Figure 3:**
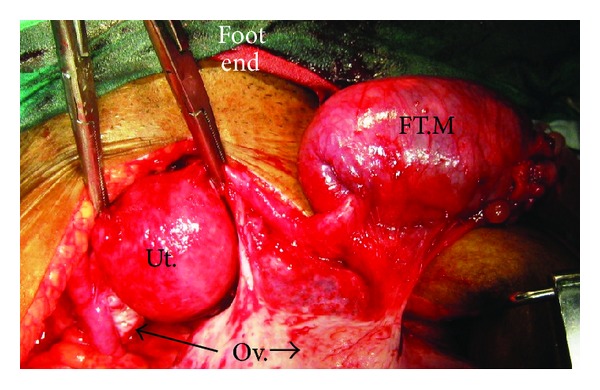
Right tubal mass (FT.M), normal bilateral ovaries (Ov.), and uterus (Ut.).

Preoperative radiological interpretations fell short of diagnosis and were reported as adnexal mass mostly ovarian in origin in 7/8 of (87.5%) women and normal in 1/8 (12.5%). The diseased tubes ranged from 7 to 15 cms in length, 3 cms to 5 cms in breadth, and 3 cms to 7 cms at the maximum point of growth and an average of 9.5 × 3.5 × 4 cms. Case 1 had only thickened bilateral tubular walls. Two women (cases 2, and 3) had soft to firm tubular masses with torsion and tubal ostia occluded due to growth, Figures 1 and 2. Case 4 did not have any torsion or growth at the fimbrial end except for an asymmetrical tubal mass, Figure 3.

Preoperative cancer antigen 125 (CA-125) was measured in 8 women and was found to be below 65 U/mL in 4 women in early stage disease (range 8.38–60 U/mL) and >65 in advanced stage (range 400 to 988 U/mL). An elevated CA-125 correlated with high stage disease Stages III and IV. One woman (case 1) had received treatment for breast cancer and one woman (case 6) had a family history of colonic carcinoma in her brother (Tables 1 and 2).

Staging laparotomy was done in the 4 clinically apparent early stages following an intraoperative diagnosis of fallopian tube cancer by frozen section.

In advanced stages, primary optimal cytoreductive surgery was achievable in 3/4 (75%) and suboptimal in 1/8 (12.5%) due to intraoperative hypotension, old age, and disease all around the base of mesentery (case no. 6—Table 1).

**Figure 4:**
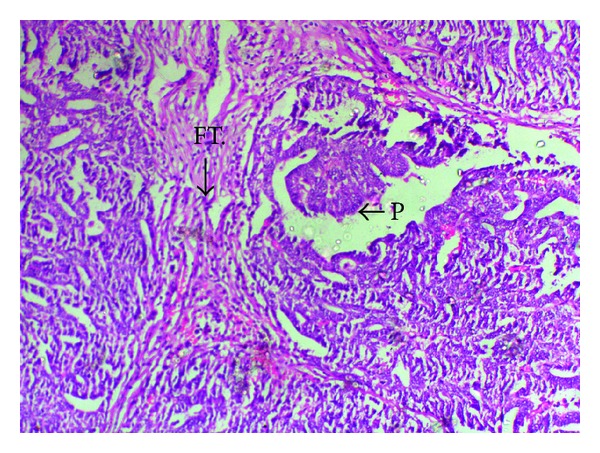
Hand E (low power) staining showing the fallopian tube wall (FT.) and growth in the cavity forming papillary projections (P).

**Figure 5:**
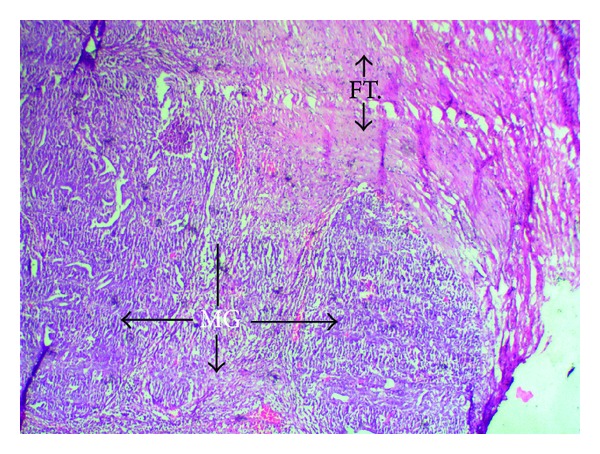
Hand E (high power) staining showing the fallopian tube wall (FT.) and growth arising from the wall and forming papillary projections due to the malignant growth (MG).

Final histopathology could categorise 4/8 (50%) of women into early stage disease (Stages IA, B, and C to Stage IIA and all grades (G1, 2, and 3) and 4/8 (50%) into advanced stage (IIB–IVB) and all grades (G1, 2, and 3), Table 1 and Figures 4 and 5.

1/8 (12.5%) had grade 2 and 7/8 (87.5%) had grade 3 tumours. Grade 3 tumours were more prevalent in advanced stage. Microscopic and macroscopic pelvic nodal disease was observed only in advanced stage disease 2/4 (50%), Tables 1 and 2.

8/8 (100%) women had received adjuvant chemotherapy. Women with early stage, that is, 4/4 (100%), have received and responded to single agent carboplatin. 4/4 (100%) in the advanced stage had received adjuvant paclitaxel and carboplatin combination (T+C), showing a 100% response to therapy, Tables 1 and 2.

**Figure 6:**
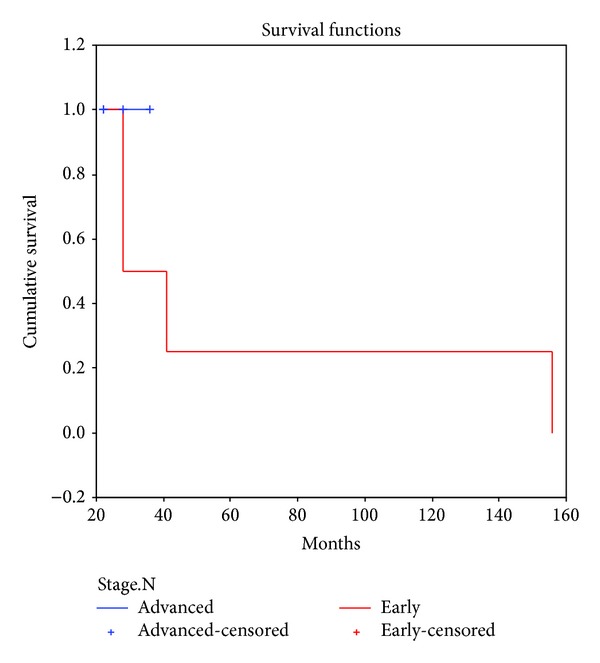
Overall survival of early stage (IA/IB/IC-IIA, all grades) and advanced stage (IIB/III/IV) PFTC women. In early stage, all women are alive. In advanced stage, only 2 were alive at the end of 24 months.

All 4/4 (100%) women in early stage disease are alive without clinical, radiological, or biochemical evidence of recurrence. 100% are alive at the end of 2 years and the longest survivor has completed 13 years (Figure 3), whereas 4/4 (100%) in advanced stage disease have recurred. Recurrence was detected by appearance of new lesions on USG or CT scan, pleural effusion detected by chest X-ray and proven cytologically. They had mean progression-free survival of 19 months (range 15–21 months) and mean overall survival of 27 months (range 22–36 months), Tables 1 and 2 and Figure 6.

## 4. Discussion

The PFTC is a rare gynaecologic malignancy with the rates reported in the literature ranging from 0.1 to 1.8% [1]. In our institution, 8 woman were identified among 7000 (0.11%) women operated between the years 2000 and 2013 for ovarian pathology.

The mean age was 55.5 years (range 40–68 years), mean parity was 2 (range 0–4), and 5/8 (62.5%) of the women were postmenopausal with a mean postmenopausal period of 6 years (2–16 years) and were comparable with the literature [18, 19].

In our series predominating symptom and the commonest clinical finding was pain with distension of abdomen and a palpable mass in 87.5%; other authors have reported an incidence of about 39–42% in their series [10, 14, 15].

The pathognomonic triad of PFTC, characterized by intermittent, profuse watery discharge, followed by relief of colicky pain abdomen and disappearance of the pelvic mass, was not found in any of our women and at the most one woman had intermittent episodes of watery vaginal discharge and some relief of abdominal pain. Authors have reported this triad to occurrence between 3–14% [6, 19].

There is a low rate of identification of PFTC preoperatively and even intraoperatively [18]. Cancer antigen 125 (CA-125) was measured in 8 women and was found to be below 65 U/mL in 4 women in early stage disease (range 8.38–60 U/mL) and >65 in advanced stage (range 400–988 U/mL). An elevated CA-125 correlated with advanced Stage III and IV. In the present series frozen section was 100% sensitive in detecting especially all the early stage women, hence intraoperative decision to proceed with staging laparotomy and thereby avoiding relaparotomy and missing advanced stage disease. However, pelvic node metastases both microscopic and macroscopic were noted in 50% of advanced stage disease. None of the early Stage IA/B/C to Stage IIA had nodal involvement and there was no upstaging as against 44.44% upstaging from Stage IC to Stage IIIC by Alvarado-Cabrero et al. [8].

Although data is limited with the use of carboplatin and paclitaxel as the first line treatment in PFTC, the last decade has witnessed the experiences shared by Gemignani et al. [20]. He reported 24 patients with advanced stage in 71%, of them 96%, 90% had overall survival at the end of 1 year and 3 years respectively. Median disease progression-free survival of 27 months for the entire population. The disease progression-free survival at 3 years was 67% in the optimally debulked group compared with 45% in the suboptimally debulked group. They concluded a possibility of excellent survival in the optimally cytoreduced patients with PFTC and adjuvant treatment with a paclitaxel-based chemotherapy regimen.

Baekelandt et al. [21] reported a 37.5% complete response, 50% partial response and 87.5% overall response when T+C was administered to 8 women with PFTC.

One of the largest series consisting of 64 women with PFTC was reported by Pectasides et al. in 2009 [7] where carboplatin and paclitaxel therapy was used in chemonaive patients. They reported a 93% response rate and an excellent 5-year survival rate.

In the present series we had 100% overall response rate to the first line T+C in all 4 women with advanced disease, but they recurred with the disease and succumbed, with mean progression-free survival of 19 months (range 15–21 months) and mean overall survival of 27 months (range 22–36 months).

The most important prognostic factors for survival appear to be the initial stage of disease [7, 15, 20] and optimal cytoreduction [15, 20, 22] as published in the literature. Similar observations were also made in the present study where all 4/4 (100%) women in early stage disease are alive without clinical, radiological, or biochemical evidence of recurrence.

## 5. Conclusion

Fallopian tube carcinoma is a rare gynaecological tumor. Early stage and optimal cytoreduction are the most significant factors in prolonging survival of women with PFTC. Paclitaxel and carboplatin or single agent carboplatin chemotherapy is a valid option in treating primary fallopian tube carcinoma. Intraoperative frozen section influences decision to perform staging laparotomy.

## Figures and Tables

**Table 1:** Details of women characteristics.

Case number	Age	Parity	Menopausal/past/family	Presenting comp.	Preop Usg/CT	Preop CA-125 U/m	Surgical proc/FS	HPR/stage	Adj.CT	Followup
1	58	P0L0	PM/breast ca/—	Pain abd. watery vaginal discharge	USG-N	15.5	TAH + BSO + TO + RPLND + PC/yes	High grade (G3) serous adeno ca./IB (both tubes)	6 C	28 M Alive
2	40	P1L1	REG/—/—	Pain abd. watery vaginal discharge	CT-ovarian cyst 9.5 × 4 × 4.3 cms	08.38	TAH + BSO + TO + RPLND + PC/yes	Poorly (G3) diff.adeno ca/IC/PC+	6 C	28 M Alive
3	47	P2L2	REG/—/—	Pain abd.	CT-adnexal mass-tubular shape 15 × 7 × 4 cms. Torsion+	16.1	TAH + BSO + TO + RPLND + PC/yes	Mod. diff. (G2) papillary.adeno ca./IC/PC+	6 C	41 M Alive
4	42	P3L2	REG/—/—	Pain abd.vaginal discharge	USG-ovarian cyst-7 × 5 × 3 cms, ? torsion	60	TAH + BSO + TO + RPLND + PC/yes	Poorly diff. (G3) adeno ca/IIA/ext. Ut.	6 C	156 M Alive
5	56	P2L2	PM/—/—	Pain and distension abd.	USG-adv. ovarian ca.	>400	TAH + BSO + TD + TO + PLND + appendicectomy + RS-RA PC/NO/OCS	High grade (G3) serous adeno carcinoma/IIIC/pelvic nodes+	6 T + C	PFS-20 M and died at 28 M
6	68	P4L4	PM/—/brother colonic ca	Pain and mass abd.	USG: adv. ovarian ca. asicitic+	986	TAH + BSO + TD + TO + PLND + pelvic peritonectomy + PC/NO (SODS)	Poorly diff. (G3) adeno carcinoma/IIIC/both nodes+	6 T + C	PFS-15 M Died at 22 M
7	55	P0L0	PM/—	Pain and mass abd.	USG: adv. ovarian ca. asicitic+	584	TAH + BSO + TD + TO + PLND + DS/NO/OCS	Poorly diff. (G3) adeno ca./IIIA/ micrometastasis to the omentum	6 T + C	PFS-18 M Died at 22 M
8	59	P4L4	PM/—	Pain and mass abd.	USG: adv. ovarian ca. ascitis.	988	TAH + BSO + TD + TO + RPLND + DS/NO/OCS	High grade (G3) serous adeno carcinoma/IIB	6 T + C	PFS- 21 M Died at 36 M

P: parity; L: living children; his.: history; PM: postmenopausal; comp.: complaints; USG: abdominopelvic ultra sound; Adj. CT: adjuvant chemotherapy; Adv.: advanced; ca.: carcinoma; TAH: total abdominal hysterectomy; BS0: bilateral salphingooophorectomy; RPLND: retroperitoneal pelvic lymph node dissection (pelvic + para-aortic); PLND: pelvic lymphadenectomy; TD: tumour debulking; TO: total omentectomy; DS: diaphragmatic stripping; RS-RA: rectosigmoid resection and anastomosis; Proc.: procedure; T + C: taxol + carboplatin; C: carboplatin; PFS: progression-free survival; M: months; FS: frozen section; HPR: histopathology report; abd.: abdominal; G: grade; Ut.: uterus.; SODS: suboptimal debulking surgery; OCS: optima cytoreductive surgery.

**Table 2:** Summary of women characteristics and treatment.

Parameters	%
Age (mean and range)	55.5 yrs. (range 40–68 years)
Postmenopausal	62.5%
ECOG performance score	
0	50%
1	25.0%
2	12.5%
3	12.5%
Histology on frozen section in the presumed early stage group	
Serous	50%
Poorly differentiated carcinoma	50%
Final histopathology	
Serous	37.5%
Poorly differentiated carcinoma	50%
Papillary carcinoma	12.5%
Histological grade	
1	0%
2	12.5%
3	87.5%
FIGO stage	
Early stage (IA/B/C/IIA and all grades 1, 2, 3)	4/8 (50%)
Advanced stage (IIB/III/IV and all grades 1, 2, 3)	4/8 (50%)
Surgical procedure	
Staging laparotomy	4/4 (early stage) 100%
Optimal debulking surgery	3/4 (advanced stage) 75%
Suboptimal surgery	1/4 (advanced stage) 25%
Chemotherapy response	
Single agent carboplatin was administered in early stage disease	4/4 (100%)
Paclitaxel and Carboplatin combination in advanced stage disease	4/4 (100%)
